# Cost and effectiveness of prescribing emollient therapy for atopic eczema in UK primary care in children and adults: a large retrospective analysis of the Clinical Practice Research Datalink

**DOI:** 10.1186/s12895-018-0076-y

**Published:** 2018-10-29

**Authors:** George Moncrieff, Annie Lied-Lied, Gill Nelson, Chantal E Holy, Rachel Weinstein, David Wei, Simon Rowe

**Affiliations:** 1 Mayfield Clinic Summertown, Oxford, OX2 7DE UK; 2 Johnson & Johnson Ltd (UK), Maidenhead, Berkshire, UK; 3 Johnson & Johnson, Inc, New Brunswick, NJ USA; 4 Janssen Research and Development, LLC, Titusville, NJ USA; 5 NHS Wakefield Clinical Commissioning Group, West Yorkshire, UK

**Keywords:** Atopic dermatitis, Eczema, Emollient, Healthcare utilisation, Colloidal oatmeal, CPRD, Topical steroid, Antimicrobial

## Abstract

**Background:**

The Clinical Practice Research Datalink (CPRD) was used to evaluate the overall costs to the National Health Service, including healthcare utilisation, of prescribing emollients in UK primary care for dry skin and atopic eczema (DS&E).

**Methods:**

Primary care patients in the UK were identified using the CPRD and their records were interrogated for the 2 years following first diagnosis of DS&E. Data from patients with (*n* = 45,218) and without emollient prescriptions (*n* = 9780) were evaluated. Multivariate regression models were used to compare healthcare utilisation and cost in the two matched groups (age, sex, diagnosis). Two sub-analyses of the Emollient group were performed between matched groups receiving (1) a colloidal oatmeal emollient (Aveeno-First) versus non-colloidal oatmeal emollients (Aveeno-Never) and (2) Aveeno prescribed first-line (Aveeno-First) versus prescribed Aveeno later (Aveeno-Subsequently). Logistic regression models calculated the odds of prescription with either potent / very potent topical corticosteroids (TCS) or skin-related antimicrobials.

**Results:**

Costs per patient were £125.80 in Emollient (*n* = 7846) versus £128.13 in Non-Emollient (*n* = 7846) matched groups (*p* = 0.08). The Emollient group had fewer visits/patient (2.44 vs. 2.66; *p* < 0.0001) and lower mean per-visit costs (£104.15 vs. £113.25; *p* < 0.0001), compared with the Non-Emollient group. Non-Emollient patients had 18% greater odds of being prescribed TCS and 13% greater odds of being prescribed an antimicrobial than Emollient patients. In the Aveeno-First (*n* = 1943) versus Aveeno-Never (*n* = 1943) sub-analysis, costs per patient were lower in the Aveeno-First compared with the Aveeno-Never groups (£133.46 vs. £141.11; *p* = 0.0069). The Aveeno-Never group had ≥21% greater odds of being prescribed TCS or antimicrobial than the Aveeno-First group. In the Aveeno-First (*n* = 1357) versus Aveeno-Subsequently (*n* = 1357) sub-analysis, total costs were lower in the Aveeno-First group (£140.35 vs. £206.43; *p* < 0.001). Patients in the Aveeno-Subsequently group had 91% greater odds of being prescribed TCS and 75% greater odds of being prescribed an antimicrobial than the Aveeno-First group.

**Conclusions:**

Acknowledging limitations from unknown disease severity in the CRPD, the prescription of emollients to treat DS&E was associated with fewer primary care visits, reduced healthcare utilisation and reduced cost. Prescribing emollients, especially those containing colloidal oatmeal, was associated with fewer TCS and antimicrobial prescriptions.

**Trial registration:**

The study is registered at http://isrctn.com/ISRCTN91126037.

**Electronic supplementary material:**

The online version of this article (10.1186/s12895-018-0076-y) contains supplementary material, which is available to authorized users.

## Background

Dry skin and atopic eczema (DS&E), also described as atopic dermatitis (AD), is a common condition characterised by inflammatory flares followed by periods of remission. Prevalence estimates vary across diagnosis codes, age, and country, but in the UK, a range from 6 to 34% seems probable [1, 2].

A 1998 analysis within the UK National Health Service (NHS) estimated the cost of treatment of DS&E to be over £100 million each year [3]. Costs since then are likely to have increased significantly as the number of eczema-related UK general practitioner (GP) visits increased from 3.77 to 4.02 per person per year and the number of eczema-related prescriptions increased 56.6% from 2001 to 2005 [4]. Flares may occur frequently (as often as two or three times per month) and can have a negative effect on quality of life [5]. In one study, individuals with AD on average reported having over nine flares a year, with flares lasting around 2 weeks [6].

Treatment of DS&E typically includes use of emollients, which reduce the number of flares, prolong the interval between flares, and reduce the need for topical corticosteroids (TCS) [7–12]. Emollients were recommended for use as first-line therapy for patients with DS&E by a recent expert consensus group [7].

Evidence also suggests benefits of emollients in avoiding the development of AD. AD is often the first indication of the “atopic march,” a progression to other diseases (food allergy, asthma, and allergic rhinitis) that can appear later in life in affected individuals [13]. In a US/UK study in a population of neonates at high risk for developing AD, use of emollients beginning within 3 weeks of birth for 6 months reduced the risk of developing AD by 50% [14], and investigations continue with a large, ongoing clinical trial (Barrier Enhancement for Eczema Prevention [BEEP]) to evaluate the effectiveness of emollient therapy during the first year of life [15]. In a pilot study, use of an emollient for the first 6 months of life was associated with a trend towards reduced AD and food sensitisation at 1 year of age [16]. In Japanese infants at high risk for developing AD, use of emollients from birth to 32 weeks of life reduced the risk of developing AD by 32% [17].

In England, the National Institute for Health and Care Excellence (NICE) provides clinical guidance on the management of atopic eczema and cost-effective treatment options, during and between flares of the condition, in children under 12 years of age. The treatment strategy for atopic eczema is a stepped approach with an emollient prescribed at each step, regardless of the severity of the condition [5]. Although the NICE guidance applies only to children, current advice from the Primary Care Dermatology Society also recommends a similar stepwise approach for the management of atopic eczema in adults [17].

Adding emollients to the treatment of children with DS&E was cost-neutral in prior studies — the added cost of the emollient being offset by a reduction in other treatment costs for DS&E [18–20]. However, despite acceptance by expert clinical groups [21, 22], the value and effectiveness of prescribing some emollients remain to be proven.

In this study, the Clinical Practice Research Datalink (CPRD) database [23] was interrogated to analyse emollient use and healthcare utilisation in patients with DS&E beginning in 2008—a year after publication of NICE guidelines recommending emollient therapy [5, 24]. In addition, our study investigated the use of Aveeno, an emollient that contains active colloidal oatmeal known to be beneficial in the treatment of DS&E [25, 26].

## Methods

This was a retrospective group study using data from the CPRD database of anonymised patient medical records. The study protocol (# 16_198R) was approved by the Independent Scientific Advisory Committee (ISAC) for the Medicines & Healthcare Products Regulatory Agency and patient-informed consent was not required for use of this observational dataset. No additional institutional review board approval is required for CPRD studies. The study is registered at http://isrctn.com/ISRCTN91126037.

### Group identification

All patients with a DS&E diagnosis code (see Additional file 1: Diagnosis codes) between 2008 and 2012 diagnosed during a regular primary care consultation were identified. The date of the first DS&E diagnosis was referred to as “index” diagnosis.

**Fig. 1:**
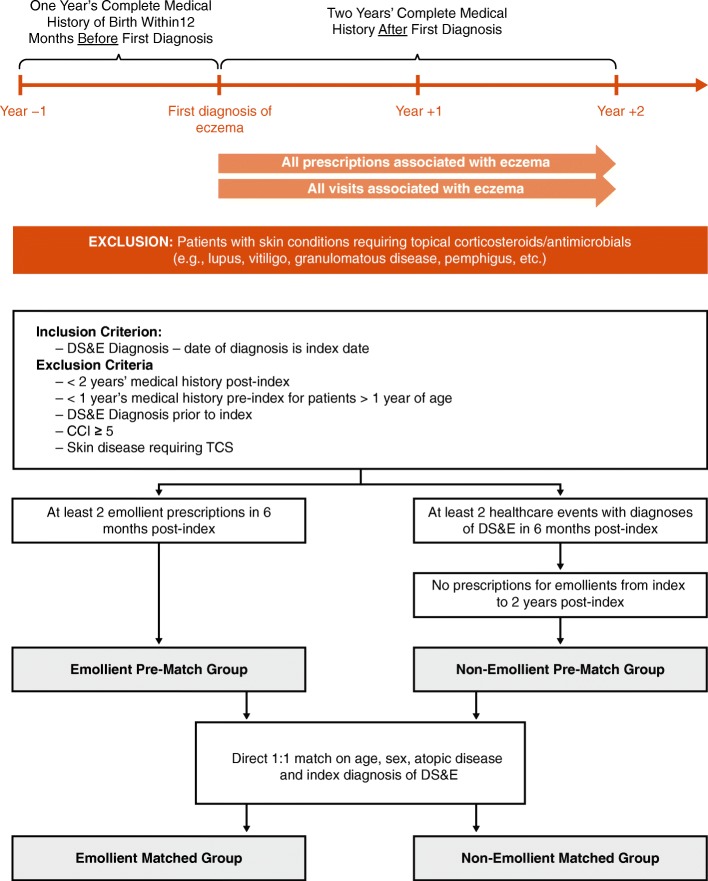
Study design (top) and group identification algorithm (bottom)

The group comprised (1) all patients aged 1 year and older at index who had at least 12 months pre- and 2 years post-index complete medical history, and (2) patients less than 1 year of age at index who had complete medical records in the database from birth to at least 2 years post-index. Patients with any diagnosis of DS&E during the period before index (“washout window”) were excluded, thus ensuring as far as possible that all patients had their first diagnosis of DS&E at time of index. The study period started at index and continued for 2 years post-index (Fig. 1).

The period before index was used to analyse presence of comorbidities. Comorbidities analysed during the pre-index period included atopic diseases (asthma, food allergies, and allergic rhinitis) often associated with the “atopic march” [13]. The Charlson Comorbidity Index (CCI), a widely used index originally developed to predict mortality based on the presence of 19 conditions, was also evaluated. The CCI can be used to study burden of disease and is indicative of overall health status [27–29]. All patients with a CCI ≥5 were excluded from the study as it was determined that their significant comorbidities may act as confounders in the analyses.

During the pre-index and study period, all patients’ diagnoses were analysed for coincident skin diseases that could result in significant use of TCS, such as bullous pemphigoid, lupus, lichen planus, and vitiligo (see Additional file 2). Patients with any such diagnosis before or during the study period were excluded from the study, as it might have been difficult to distinguish use of TCS for these conditions versus for DS&E.

From this population, patients with at least two distinct emollient prescriptions within 6 months of index were identified and included in the “Emollient” group. Of the remaining patients, those with at least two healthcare visits with diagnoses of DS&E within 6 months of index but no emollient prescriptions at any time from index to 2 years post-index were included in the “Non-Emollient” group.

These “Emollient” and “Non-Emollient” groups were further defined as the “Pre-match” groups. Pre-matched groups were then matched using a 1:1 exact matching algorithm based on age, presence of AD (defined as having at least one of the following conditions: food allergy [Y/N], allergic rhinitis [Y/N], asthma [Y/N]), sex, and index diagnosis. The inclusion of index diagnosis in the variables used for matching was particularly important as different index diagnoses may have reflected slightly different disease severities and presentation. Using a direct match with index diagnosis as a variable ensured both investigational and control arms of having similar diagnoses at index. The final cohorts were defined as matched Emollient and matched Non-Emollient groups. A graphical representation of the group identification methodology is shown in Fig. 1.

### Outcomes identification

The following outcomes were identified for all patients: (1) Frequency and cost of visits with DS&E diagnoses; (2) total cost of prescriptions provided during visits with DS&E diagnoses, by prescription category, from index to 2 years post-index. The prescription categories were based on version 71 of the British National Formulary chapters and included TCS, antimicrobial-containing prescriptions (topical and oral), emollients, and all other; (3) presence of at least one prescription for potent/very potent TCS provided during visits with DS&E diagnoses; and (4) presence of at least one prescription for an antimicrobial-containing medication during visits with DS&E diagnoses.

### Statistical analyses

Descriptive statistics were performed on pre-match and matched groups. Continuous variables were expressed in terms of means, medians, and standard deviations. Proportions of comorbidities in each group were assessed descriptively. Standard t tests were used to assess differences between groups.

To compare healthcare utilisation between the matched groups of Emollient versus Non-Emollient patients, a multivariate regression model (generalised estimating equations [GEE]) was employed, controlling for covariates (age, sex, CCI, and presence of atopic conditions) and adjusting for correlation between clusters, defined in this study as GP practices. GEE models are robust and are designed to cope with outcomes with different distributions, and have the capacity to adjust the correlation within clusters [30].

To estimate the odds of being prescribed either a potent or very potent TCS or an antimicrobial-containing prescription, a logistic regression model was built using all available variables. These odds were calculated for the matched Emollient versus Non-Emollient groups and the c values were captured for each model.

#### Subgroup analyses

Current guidelines recommend that patients be offered a choice of emollient to choose one agreeable to the individual [5], as emollients that feel agreeable are more likely to be used appropriately and therefore to yield better outcomes. Additional subgroup analyses were performed within the Emollient group and were designed to evaluate whether the prescription of Aveeno-branded products containing colloidal oatmeal, which have been shown previously to be well-liked by patients [31], result in lower healthcare utilisation than other emollients. Two sub-analyses were performed.

1. Aveeno-First versus Aveeno-Never: Patients from the Emollient pre-match group prescribed an Aveeno-branded emollient at time of index were identified and defined as Pre-Match “Aveeno-First.” Of the remaining patients, those who were never prescribed an Aveeno-branded emollient at any time during the study period were categorised as Pre-Match “Aveeno-Never.” The Aveeno-First and Aveeno-Never pre-match groups were matched using a direct matching algorithm as defined above and compared using the outcomes and statistics as also defined above.
2. Aveeno-First versus Aveeno-Subsequently: Patients who were prescribed an Aveeno-branded product subsequent to another emollient between days 5 and 730 post-index were categorised as pre-match “Aveeno-Subsequently,” and included those who may have received Aveeno at a later stage, for example, as a third or fourth choice. The pre-match Aveeno-First and Aveeno-Subsequently groups were matched and outcomes analysed as described above

### Cost analysis

Costs of all patient contacts were analysed and “priced” accordingly (e.g., nurse contact had a lower price than GP contact), using costs as outlined in the Personal Social Services Research Unit (PSSRU) Costs of Health and Social Care for 2015 [32]. Prescription costs were estimated using publicly available costs per drug for 2015 [33]. The net ingredient cost (NIC) was obtained for all prescriptions and linked to all prescriptions within CPRD. The total cost for all prescriptions were then calculated by taking the amounts prescribed multiplied by the NIC per quantity. The perspective of this analysis is strictly that of the UK NHS—no other societal or otherwise related costs were included in the total cost of care. All analyses were performed using SAS 9.4 (SAS Institute, Cary, NC).

## Results

### Emollient versus Non-Emollient

A total of 54,998 patients met the inclusion criteria, with 45,218 patients in the Emollient group and 9780 patients in the Non-Emollient group (Table 1). Percentages of patients with ADs and the age distribution differed significantly between groups. Table 1 shows the 13 most prevalent index diagnoses in each group. The most prevalent diagnosis in both groups was AD/eczema. Many of the differences between the groups most likely represent diseases of children versus adults, with most (55%) of the patients who received emollient prescriptions being < 16 years of age, whereas only 15% of those in the Non-Emollient group were aged < 16 years.

**Table 1:** Study population (before matching)

	Emollient (*n* = 45,218)	Non-Emollient (*n* = 9780)
Regional distribution %		
East Midlands	8.91	10.55
London	12.5	8.67
North East	1.75	3.13
North West	11.87	14.66
Northern Ireland	3.7	1.51
Scotland	10.79	9.62
South Central	11.62	13.15
South East Coast	7.95	11.13
South West	7.2	8.26
Wales	12.36	6.56
West Midlands	9.43	10.47
Yorkshire and Humber	1.92	2.27
Age distribution %		
Less than 1 year	13.43	1.17
1 to 5 years	29.24	6.13
6 to 10 years	7.1	3.21
11 to 15 years	5.64	3.64
16 to 18 years	2.87	2.32
19 to 65 years	26.38	63.11
More than 65 years	15.33	20.42
Disease type %		
Atopic dermatitis/eczema	43.62	26.24
Contact dermatitis	2.8	6.95
Dermatitis NOS	6.31	14.39
Dermatitis/dermatoses	1.49	2.84
Eczema NOS	18.36	15.12
Flexural eczema	3.37	1.44
Hand eczema	1.04	1.29
Infantile eczema	13.84	1.55
Infected eczema	2.57	4.54
Itch	2.06	7.46
Pruritus NOS	3.28	9.71
Seborrhoeic dermatitis capitis	0.45	5.62
Skin irritation	0.81	2.85

Note that responses from 1451 subjects for Disease Type were missing and not included in this analysis

NOS, not otherwise specified

#### Matching

Direct matching (1:1) to normalise differences between groups in age, presence of atopic disease, sex, and index diagnosis resulted in 7846 patients in both the Non-Emollient and the Emollient matched groups. After matching, differences remained in terms of geographic location and CCI distribution. More patients with CCI = 0 were in the Non-Emollient group versus the Emollient group; however, both groups had the same percentage of females (59.62%), similar age distribution (82.00% ≥19 years of age), the same percentage of patients with AD (allergic rhinitis: 7.18%, asthma: 13.05%, food allergies: 0.25%), and the same index diagnoses. Inter-group differences were within the designed scope of the GEE models.

#### Healthcare utilisation

**Fig. 2:**
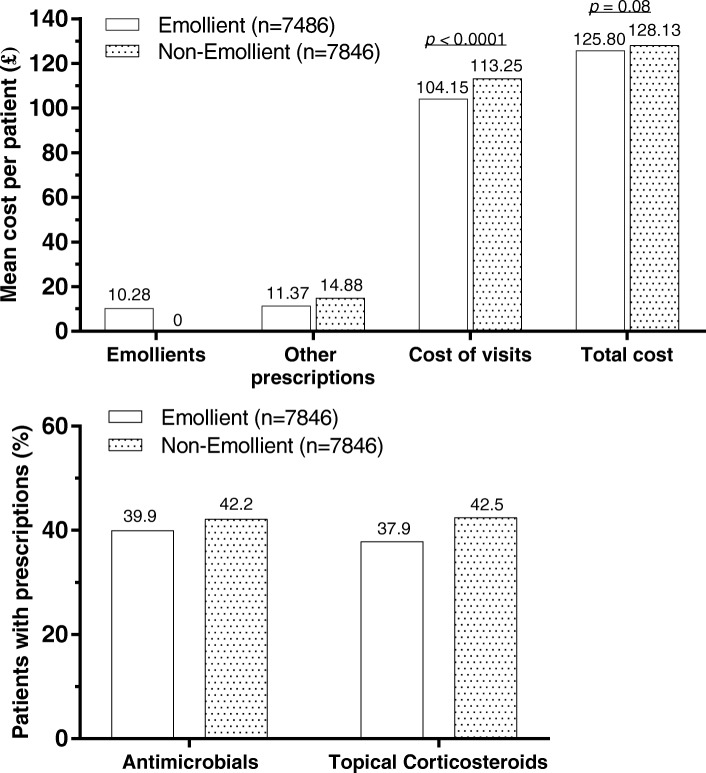
Costs (top) and medication use (bottom) in matched Emollient versus Non-Emollient groups

Costs to the NHS over the 2-year study period were approximately the same in the Emollient (£125.80) versus Non-Emollient (£128.13) matched groups, with the costs of emollients in the Emollient group offset by lower costs for visits and other prescriptions (Fig. 2). The number of visits was statistically significantly lower in the Emollient (2.44 visits) versus Non-Emollient (2.66 visits) group (*p* < 0.0001), a difference of 9.06% (95% confidence interval [CI]: 7.19–10.97%). The decreased number of primary care visits translated into a significantly decreased overall cost of visits for the Emollient group (£104.15 vs. £113.25 for Non-Emollient, *p* < 0.0001). The difference was estimated at 8.74% (95% CI: 6.96–10.56%). Using the GEE model, total cost of care to the NHS was estimated at £125.98 per patient in the Emollient group versus £127.98 in the Non-Emollient group. The difference suggested a non-statistically significant trend (*p* = 0.08) of 1.58% of increased costs for those in the Non-Emollient group.

Prescribing an emollient was associated with reduced prescriptions of potent or very potent TCS in the matched groups (Fig. 2). The proportion of patients treated with potent/very potent TCS was 42.45% in the Non-Emollient group compared with 37.89% in the Emollient group, and the odds of being prescribed a potent or very potent TCS in the Non-Emollient group was 1.18 (95% CI: 1.10–1.26), indicating an 18% increased odds of treatment compared with the reference Emollient group.

The proportion of patients treated with antimicrobial-containing prescriptions was 42.19% (3310/7846) in the Non-Emollient group compared with 39.96% (3135/7846) in the Emollient group (Fig. 2). The odds of being prescribed an antimicrobial-containing prescription in the Non-Emollient group was 1.13 (95% CI: 1.06–1.21), suggesting a 13% increased odds of treatment versus patients in the reference Emollient group.

### Aveeno-First versus Aveeno-Never

Matched groups of 1943 patients each for the Aveeno-First versus Aveeno-Never groups were analysed.

**Fig. 3:**
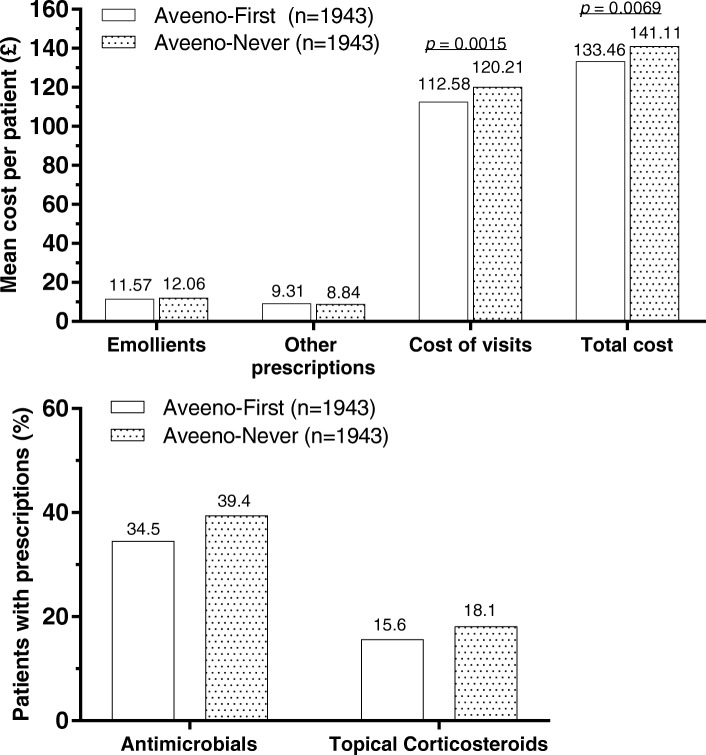
Cost (top) and medication use (bottom) in matched Aveeno-First versus Aveeno-Never groups

Costs for emollient and/or non-emollient prescriptions did not differ between Aveeno-First and Aveeno-Never groups (Fig. 3). Additionally, patients in the Aveeno-First group made fewer visits than matched patients in the Aveeno-Never group (2.68 vs. 2.83 visits; *p* = 0.0081), resulting in lower visit costs and statistically significantly lower overall costs (*p* = 0.0069) in the Aveeno-First group.

The percentages of patients treated with potent/very potent TCS were lower in the Aveeno-First (15.64%) versus Aveeno-Never group (18.11%; Fig. 3). The odds ratio of being prescribed a potent or very potent TCS in the Aveeno-Never versus Aveeno-First group was 1.214 (95% CI: 1.007–1.464), suggesting the Aveeno-First group was less likely to receive a prescription for a potent or very potent TCS within 2 years after the diagnosis.

The percentage of patients prescribed skin-condition-related antimicrobial was lower in the Aveeno-First (34.53%) versus Aveeno-Never group (39.42%; Fig. 3). The odds ratio of being prescribed an antimicrobial-containing prescription in the Aveeno-Never versus Aveeno-First group was 1.252 (95% CI: 1.090–1.437), suggesting the Aveeno-First group was 25% less likely to receive a prescription for a skin condition-related antimicrobial within 2 years after diagnosis.

### Aveeno-First versus Aveeno-Subsequently

**Fig. 4:**
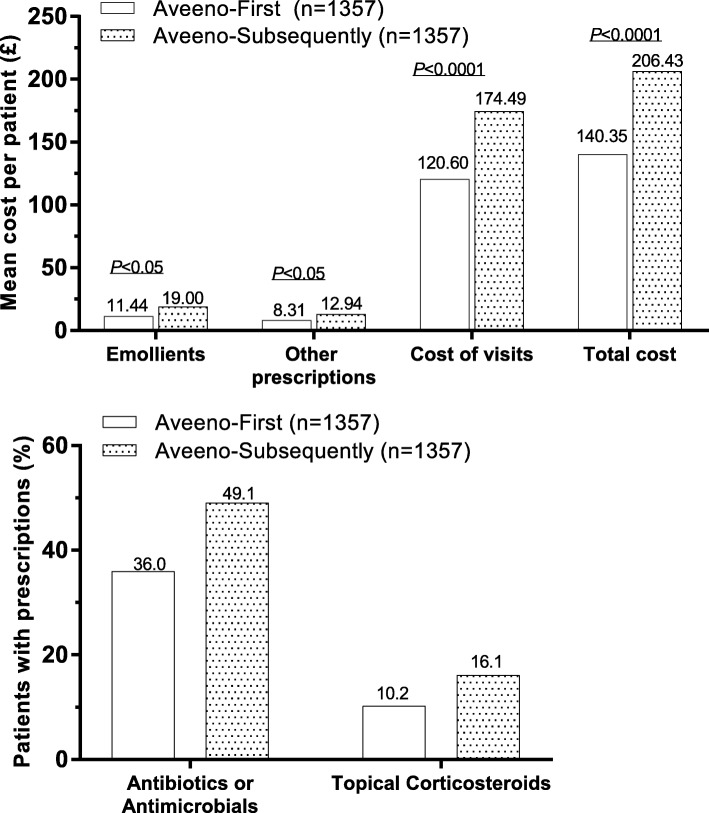
Cost (top) and medication use (bottom) in matched Aveeno-First versus Aveeno-Subsequently groups

The second sub-analysis included 1357 matched patients in each group, namely the Aveeno-First and the Aveeno-Subsequently groups. Delayed prescribing of Aveeno was associated with significantly higher costs for prescriptions and more GP visits within the 2 years following the diagnosis of atopic eczema (2.89 visits for Aveeno-First vs. 4.14 visits for Aveeno-Subsequently, a difference of 43.1%). Prescribing Aveeno as a first-line treatment for DS&E was associated with lower visit costs and lower overall costs than in the Aveeno-Subsequently group within the 2 years following the index diagnosis (Fig. 4).

The percentage of patients prescribed potent or very potent TCS (Fig. 4) was lower in the Aveeno-First group (10.24%) than in the Aveeno-Subsequently group (16.14%), with the odds ratio of being prescribed a potent or very potent TCS in the Aveeno-Subsequently group versus the Aveeno-First group being 1.914 (95% CI: 1.489–2.462). Hence, patients had 91% greater odds of receiving a prescription for a treatment with potent or very potent TCS when not prescribed Aveeno first than when Aveeno was prescribed first.

The percentage of patients treated with skin-condition-related antimicrobials was also lower in the Aveeno-First group (35.96%) than in the Aveeno-Subsequently group (49.08%; Fig. 4). The odds ratio of being prescribed an antimicrobial in the Aveeno-Subsequently group versus the Aveeno-First group was 1.748 (95% CI: 1.495–2.044). Hence, patients who did not receive Aveeno first-line had 75% greater odds of treatment with antimicrobial than when Aveeno was received first-line.

## Discussion

This study evaluated the utilisation and costs of primary healthcare resources using data from the CPRD to estimate the value of prescribing emollients for DS&E to patients presenting to their GP in the UK. In examining the component costs of care, including emollients, other medicines prescribed for DS&E, and patient visits, the most important cost driver appeared to be the number and associated cost of patient visits by patients to primary care, with the Emollient and Aveeno-First groups having fewer clinic visits and therefore lower overall costs than the Non-Emollient and Aveeno-Never groups. Reducing repeat visits is particularly relevant to the UK, where the annual GP consultation rate per person increased more than 13% from 2007 to 2014 [34], and has continued to increase to the point that GP practice is in a state of crisis with escalating demand outstripping capacity [35, 36].

Skin disorders, such as eczema, make up a significant portion of the GP’s workload in the UK and elsewhere [19, 37], and primary care visits in the UK for skin diseases and eczema have been increasing year on year [4]. A 2014 King’s Fund report [38], using data based on a report by Schofield et al. [39], suggested that, with an average of two consultations per episode, the average GP with a patient list size of 1700 would have had 630 consultations for skin conditions per year in 2006 [39]. Reducing patient visits to primary care would not only benefit patients and reduce demand on primary care, but could also help reduce the burden on the entire healthcare service [36, 40].

This study suggests that a policy to prescribe emollients to patients when the diagnosis of DS&E is initially made may save the NHS money, or at least be cost-neutral. Initial analysis identified that more patients with a diagnosis of DS&E received a prescription for an emollient than those who did not (45,218 vs. 9780). The age distribution of Emollient versus Non-Emollient pre-match groups indicated that the Emollient group was substantially younger than the Non-Emollient group. As prescriptions for children under 16 years are dispensed free of charge, they may be more likely to be filled than those incurring a cost to the patient. Hence, non-paediatric patients may be encouraged to purchase an emollient rather than fill a prescription, and thus would incur personal costs to obtain treatment for this condition that would not be recorded in the CPRD. The effect of the patient purchasing an emollient under these conditions would have skewed healthcare system costs away from emollient prescriptions that were reimbursed and reduced the apparent efficacy of treatment and the observed differences seen between Emollient and Non-Emollient groups.

Although emollients are recommended as first-line therapy for DS&E [5, 7, 24], current and future cost pressures on the NHS are likely to put more pressure on primary care providers to avoid issuing prescriptions for emollients [7] or to choose the prescribed emollient primarily on the basis of cost [41]. Furthermore, patients tend to use emollients sub-optimally in insufficient quantity, and too infrequently [7, 9]. Encouraging purchase rather than prescription could exacerbate this tendency and the negative impact on clinical outcomes, potentially increasing health costs for follow-up visits and additional medications in the longer term.

In the Aveeno-First versus Aveeno-Subsequently comparison, visit and prescription costs were significantly greater when the patient did not receive Aveeno as a first-line treatment, suggesting that costs increase when a patient requires repeat healthcare visits to adjust treatment. In DS&E, this study suggests that, rather than reserving Aveeno as a second or third-line therapy, prescribing Aveeno first-line may save NHS primary care costs. In a recent UK study of emollient therapy for eczema in children, four different emollients, including Aveeno, were evaluated [20]. In that study, no significant difference was noted among the costs of the emollients, and similar to the study reported herein, the main cost driver was the cost of a primary care appointment.

This study also indicates a reduction in steroid prescriptions in the Emollient and Aveeno-First groups, and supports previous research suggesting emollient use can reduce the need for a TCS prescription [8–10, 12].

Another important finding was that emollient therapy, and Aveeno in particular, might reduce the need for prescriptions of antimicrobial therapy in eczema. This study appears to be the first report suggesting that emollient therapy can reduce the need for antimicrobial prescriptions, and supports the findings of Francis and co-workers, who reported that oral and topical antibiotics had little or no benefit to reduce eczema severity in children with clinically infected eczema already being treated with emollients and TCS [42, 43]. Reducing antibiotic use will help the GP professional community to contribute to antimicrobial stewardship goals [44, 45], thereby slowing the development of antimicrobial resistance, an area of grave concern [46]. Additional research will be required to confirm these findings.

### Limitations

As with all database studies, the findings presented herein assume accurate diagnoses of patients and do not correct for potential errors in coding. Disease types included for initial cohort identification were broad (Table 1); however, the analysis was based on groups matched exactly for index diagnosis. Furthermore, this analysis may be relevant to the UK healthcare system only, as other healthcare systems, such as those in the EU or USA, may not reimburse emollient prescriptions for DS&E and costs will be different in these healthcare systems.

Severity of disease was not available in the CPRD as disease severities are not recorded in the database beyond those captured by normal diagnoses. More severe patients prescribed a TCS may not receive a prescription for an emollient that visit because the prescriber is focussed on using the TCS. Thus, should cases in the Emollient groups (including Aveeno groups) have been less severe, this may have overestimated the effect of the emollient therapy in a group expected to have fewer visits and lower costs. However, we tried to minimise the effect of a severity bias by matching for age and type of disease.

Total costs may have been underestimated because only costs associated with a DS&E diagnosis were considered for this analysis. Personal purchase of emollients by patients was not captured in the CPRD and thus might result in an underestimate of the use of emollients and inaccurate assignment of patients to the Non-Emollient group, which would tend to bias the results towards no effect.

Conversely, prescriptions that were written but not filled cannot be identified and could have resulted in an overestimate of the proportion of emollient users, although some of those with unfilled prescriptions may have selected to purchase an emollient for themselves instead. We tried to reduce the impact of this limitation by requiring two distinct prescriptions for the Emollient group, ensuring that these patients were highly likely to have filled at least one prescription. Each of these scenarios could have influenced the study to reduce the apparent positive impact of prescribing emollients and specifically Aveeno.

Direct matching of groups and age differences between Non-Emollient and Emollient groups may have resulted in groups that do not reflect the overall population of patients with DS&E in the CPRD. In the matched group analysis of the Emollient versus Non-Emollient group, to maximise the size of the Non-Emollient group, the Emollient group had more adults than the overall pre-matched Emollient group. However, in the analyses of Emollient groups, the demographics more closely match those in the overall Emollient population.

## Conclusions

In conclusion, this study suggested that prescription of an emollient to treat DS&E may be associated with reduced cost of care, primarily owing to fewer primary care visits and other prescriptions, though disease severity is an unknown and potential confounder in this analysis. Emollient cost was a small fraction of the overall cost of treating eczema in general practice. Furthermore, prescribing emollients was associated with a potential for fewer antibiotic or potent/very potent TCS prescriptions. Timely prescribing of Aveeno to improve the integrity of the skin barrier [47, 48] can result in fewer flares and would probably result in decreased antimicrobial prescribing in UK primary care. Prescribing Aveeno was also associated with overall lower costs compared with prescribing other emollients, and overall costs were lowered most when Aveeno was prescribed first, rather than when prescribed as a second or subsequent choice for patients who may have found other products unsuitable.

## Additional files

Additional file 1: Diagnosis codes. List of diagnosis codes included in this study. (DOCX 16 kb)

Additional file 2: Exclusion diagnoses. List of diagnosis codes that were excluded from this study. (DOCX 19 kb)

## Abbreviations

AD: Atopic dermatitis
CCI: Charlson Comorbidity Index
CPRD: Clinical Practice Research Datalink
DS&E: Dry skin and atopic eczema
GEE: Generalised estimating eqs.
GP: General practitioner
ISAC: Independent Scientific Advisory Committee
NHS: National Health Service
NIC: Net ingredient cost
NICE: National Institute for Health and Care Excellence
PSSRU: Personal Social Services Research Unit
TCS: Topical corticosteroids
