# Supplementary material for: Cost and effectiveness of prescribing emollient therapy for atopic eczema in UK primary care in children and adults: a large retrospective analysis of the Clinical Practice Research Datalink
Source: BMC Dermatol. 2018 Oct 29;18:9. doi: 10.1186/s12895-018-0076-y (PMC6206824; doi:10.1186/s12895-018-0076-y)
Supplement: Supplementary file 1 — Diagnosis codes. List of diagnosis codes included in this study. (DOCX 16 kb) [file 12895_2018_76_MOESM1_ESM.docx]

**Additional file 1** Dry skin-eczema diagnoses included in the study

| Medcode | Read_Code | Description |
| --- | --- | --- |
| 1741 | M111.00 | Atopic dermatitis/eczema |
| 9041 | M12z100 | Eczema NOS |
| 5353 | M12z000 | Dermatitis NOS |
| 9041 | M12z200 | Infected eczema |
| 9041 | M113.00 | Flexural eczema |
| 5353 | M12..11 | Contact dermatitis |
| 13392 | M101.00 | Seborrhoeic dermatitis |
| 9041 | M112.00 | Infantile eczema |
| 13392 | M101.12 | Seborrhoeic eczema |
| 5353 | M1...11 | Dermatitis/dermatoses |
| 13392 | M28..00 | Urticaria |
| 13392 | M101.11 | Seborrhoeic dermatitis capitis |
| 9041 | M110.13 | Nappy rash |
| 9041 | M12z300 | Hand eczema |
| 13392 | M18z.00 | Pruritus NOS |
| 9041 | M18z.12 | Itch |
| 9041 | M07z.14 | Infected dermatitis |
| 13392 | M18z.11 | Skin irritation |
| 9041 | M102.00 | Infectious eczematoid dermatitis |
| 5353 | L30.9 | Dermatitis, unspecified |
| 9041 | M12z111 | Discoid eczema |
| 5353 | M12..12 | Contact eczema |
| 5353 | M12..00 | Contact dermatitis and other eczemas |
| 9041 | M129.00 | Irritant contact dermatitis |
| 9041 | M110.00 | Napkin dermatitis |
| 13392 | M102.11 | Pustular eczema |
| 13392 | M127300 | Photodermatitis |
| 9041 | M12z400 | Erythrodermic eczema |
| 5353 | M110000 | Candidal nappy rash |
| 8884 | M1y0.00 | Nummular dermatitis |
| 9888 | M116.00 | Neurodermatitis - diffuse |
| 9041 | M1y2.00 | Gravitational eczema |
| 5353 | M12zz00 | Contact dermatitis NOS |
| 9041 | M281.00 | Idiopathic urticaria |
| 5353 | M12z.00 | Contact dermatitis NOS |
| 8884 | L20.8 | Other atopic dermatitis |
| 9041 | L30.3 | Infective dermatitis |
| 9041 | M1B..11 | Juvenile plantar dermatitis |
| 8884 | M2...00 | Other skin and subcutaneous tissue disorders |
| 1594 | M283.00 | Dermatographic urticaria |
| 5353 | M285.00 | Cholinergic urticaria |
| 13392 | L29.9 | Pruritus, unspecified |
| 13392 | L50.9 | Urticaria, unspecified |
| 9041 | M10..00 | Erythematosquamous dermatosis |
| 9041 | M118.00 | Infantile seborrhoeic dermatitis |
| 13378 | M12y012 | Perfume contact dermatitis |
| 5353 | M12yz00 | Contact dermatitis: specified agent NOS |
| 13392 | M1z..00 | Skin and subcutaneous tissue inflammatory disorders NOS |
| 9041 | M282111 | Heat urticaria |
| 1869 | M28y.00 | Other specified urticaria |
| 23645 | M28yz00 | Other specified urticaria NOS |
| 13392 | M28z.00 | Urticaria NOS |
