# Supplementary material for: Cost and effectiveness of prescribing emollient therapy for atopic eczema in UK primary care in children and adults: a large retrospective analysis of the Clinical Practice Research Datalink
Source: BMC Dermatol. 2018 Oct 29;18:9. doi: 10.1186/s12895-018-0076-y (PMC6206824; doi:10.1186/s12895-018-0076-y)
Supplement: Supplementary file 2 — Exclusion diagnoses. List of diagnosis codes that were excluded from this study. (DOCX 19 kb) [file 12895_2018_76_MOESM2_ESM.docx]

**Additional file 2** Exclusion diagnoses

| Medcode | Read_Code | Description |
| --- | --- | --- |
| 43328 | F4E5311 | Vitiligo of eyelid |
| 975 | M295100 | Vitiligo |
| 44095 | F371000 | Polyneuropathy in disseminated lupus erythematosus |
| 106086 | ZRq8.11 | SLAM - Systemic lupus activity measure |
| 38264 | 43ac.00 | Lupus inhibitor activity |
| 28952 | AD53000 | Lupus pernio |
| 7871 | N000.00 | Systemic lupus erythematosus |
| 45726 | ZRq9.00 | Systemic lupus erythematosus disease activity index |
| 37492 | A170300 | Tuberculosis - lupus NOS |
| 14478 | 43GE.00 | Lupus anticoagulant screen |
| 101433 | N000600 | Cerebral lupus |
| 65391 | M154300 | Lupus erythematosus nodularis |
| 2667 | M154100 | Discoid lupus erythematosus |
| 67637 | A170000 | Tuberculosis - lupus exedens |
| 22205 | K01x411 | Lupus nephritis |
| 25390 | M154700 | Subacute cutaneous lupus erythematosus |
| 46675 | A170.11 | Lupus - tuberculous |
| 31564 | H57y400 | Lung disease with systemic lupus erythematosus |
| 11920 | N000400 | Systemic lupus erythematosus with pericarditis |
| 47047 | ZR2l.11 | BILAG - British isles lupus assessment group score |
| 20007 | N000000 | Disseminated lupus erythematosus |
| 16367 | A170100 | Tuberculosis - lupus vulgaris |
| 14471 | 43GD.00 | Lupus circulating anticoagulant index |
| 42719 | N000z00 | Systemic lupus erythematosus NOS |
| 99435 | N000500 | Neonatal lupus erythematosus |
| 4125 | M154.00 | Lupus erythematosus |
| 58706 | Nyu4300 | [X]Other forms of systemic lupus erythematosus |
| 36942 | N000200 | Drug-induced systemic lupus erythematosus |
| 63955 | M154600 | Lupus erythematosus unguium mutilans |
| 33449 | M154000 | Lupus erythematosus chronicus |
| 29519 | N000300 | Systemic lupus erythematosus with organ or sys involv |
| 94751 | F4D3300 | Eyelid discoid lupus erythematosus |
| 51798 | ZRq8.00 | Systemic lupus activity measure |
| 40797 | M154200 | Lupus erythematosus migrans |
| 30919 | 43c3.00 | Lupus anticoagulant screening test |
| 103784 | 42jG000 | Lupus insensitive activated partial thromboplastin time |
| 47672 | K01x400 | Nephrotic syndrome in systemic lupus erythematosus |
| 7522 | M154z00 | Lupus erythematosus NOS |
| 46148 | M154400 | Lupus erythematosus profundus |
| 44984 | M154500 | Lupus erythematosus tumidus |
| 100692 | Myu7800 | [X]Other local lupus erythematosus |
| 1621 | M170.00 | Lichen planus |
| 67900 | M170700 | Lichen planus obtusus |
| 38610 | M170100 | Lichen planus annularis |
| 43358 | M170900 | Follicular lichen planus |
| 53843 | Myu3200 | [X]Other lichen planus |
| 40455 | M170500 | Lichen planus linearis |
| 57469 | M170800 | Subacute active lichen planus |
| 18954 | M170200 | Lichen planus atrophicus |
| 7983 | M170400 | Lichen planus hypertrophicus |
| 43912 | M170000 | Lichen planus actinicus |
| 22154 | M170300 | Lichen planus bullosus |
| 38416 | M170z00 | Lichen planus NOS |
| 37255 | M15yz11 | Granuloma annulare |
| 1168 | M15y500 | Granuloma annulare |
| 4941 | M151000 | Erythema annulare |
| 101399 | M151A00 | Erythema annulare centrifugum |
| 32219 | M144300 | Foliaceous pemphigus |
| 34601 | 43mc.00 | Pemphigoid antibody level |
| 85987 | 68E0.00 | Pemphigus/pemphigoid screening |
| 49284 | PH33111 | Benign familial chronic pemphigus |
| 17810 | M142.11 | Juvenile pemphigoid |
| 17808 | M145100 | Benign pemphigus NOS |
| 45463 | M146100 | Ocular pemphigoid |
| 97478 | Myu1000 | [X]Other pemphigus |
| 70199 | Myu1200 | [X]Other pemphigoid |
| 37617 | F4Cy100 | Ocular pemphigoid |
| 67527 | M144700 | Wildfire pemphigus |
| 106167 | M144800 | Drug-induced pemphigus |
| 49282 | M144200 | Erythematous pemphigus |
| 58079 | M146z00 | Benign mucous membrane pemphigoid NOS |
| 2646 | M145.00 | Pemphigoid |
| 71937 | M146000 | Benign mucous membrane pemphigoid with no eye involvement |
| 52474 | M144000 | Benign pemphigus |
| 25738 | M144600 | Pemphigus vulgaris |
| 15415 | M144.00 | Pemphigus |
| 40275 | M146011 | Cicatricial pemphigoid |
| 34602 | 43mb.00 | Pemphigus antibody level |
| 53763 | M144z00 | Pemphigus NOS |
| 37532 | M146.00 | Benign mucous membrane pemphigoid |
| 9880 | M145000 | Bullous pemphigoid |
| 57451 | M144500 | Pemphigus vegetans |
| 58116 | M145z00 | Pemphigoid NOS |
| 685 | M240100 | Alopecia areata |
| 53782 | Myu6200 | [X]Other alopecia areata |
| 8884 | M10..00 | Erythematosquamous dermatosis |
| 653 | M101.12 | Seborrhoeic eczema |
| 3524 | M140.00 | Dermatitis herpetiformis |
| 24942 | M141.00 | Subcorneal pustular dermatosis |
| 53839 | M141.11 | Sneddon - Wilkinson disease |
| 50627 | M142.00 | Juvenile dermatitis herpetiformis |
| 33525 | M145200 | Senile dermatitis herpetiformis |
| 31667 | M145300 | Acquired epidermolysis bullosa |
| 84401 | M147.00 | Erosive pustular dermatosis of the scalp |
| 36605 | M14y.00 | Other specified bullous dermatoses |
| 14951 | M14z.00 | Bullous dermatoses NOS |
| 5287 | M152.11 | Nodular vasculitis |
| 1075 | M182.00 | Prurigo |
| 3696 | M183200 | Lichen simplex |
| 1578 | M252100 | Pompholyx unspecified |
| 900 | M252200 | Cheiropompholyx |
| 25178 | M252300 | Podopompholyx |
